# A modular digital method for eliciting multi-actor demand to prioritize agrifood research and scaling investments

**DOI:** 10.1016/j.mex.2026.104220

**Published:** 2026-10-19

**Authors:** Sreejith Aravindakshan, Timothy J. Krupnik, Dyutiman Choudhary, Shreya Chakraborty, Mustafa Kamal, Md. Harun-Or-Rashid, Gerald Atampugre, Olufunke Cofie, Paswel Marenya, Deissy Martínez-Barón

**Affiliations:** a International Maize and Wheat Improvement Center (CIMMYT), House 10/B, Road 53, Gulshan-2, Dhaka, 1212, Bangladesh; b CGIAR System Organization, House #10/B, Road #53, Gulshan-2, Dhaka, 1212, Bangladesh; c International Maize and Wheat Improvement Center (CIMMYT), NARC Research Station, Khumaltar, Lalitpur, Kathmandu, 5186, Nepal; d International Water Management Institute (IWMI), House 07, Road 56, Gulshan-2, Dhaka, 1212, Bangladesh; e International Water Management Institute (IWMI), PMB CT 112, Cantonments, Accra, Ghana; f International Maize and Wheat Improvement Center (CIMMYT), ICRAF House, United Nations Avenue, Gigiri 1041-00621, Village Market, Nairobi, Kenya; g Alliance Bioversity–CIAT, Km 17 Recta Cali–Palmira, A.A. 6713, C.P. 763537, Palmira, Valle del Cauca, Colombia

**Keywords:** Agrifood innovation systems, Multi-actor demand, Research prioritization, Innovation scaling, Large language models, Rank-based elicitation, KoboToolbox

## Abstract

Agrifood research and scaling organizations seek to be demand responsive, yet decision-makers lack a consistent method for determining whose demand counts and which innovations merit investment. We present a modular digital method combining a common survey architecture with country-specific content. Ten tools were developed for Bangladesh, Colombia, Egypt, Ethiopia, Guatemala, India, Kenya, Nepal, Nigeria, and Vietnam across CGIAR’s six regions. Each covers broad innovation domains, nested specific innovations, barriers, and enabling conditions while retaining a common analytical framework. The method profiles seven actor families and characterizes their positions within agrifood innovation systems, including their influence, while eliciting ordinal priorities. Outputs comprise stakeholder profiles and positioning, broad and nested innovation priority scores, barrier profiles, and enabling-condition profiles. Field applications comprise Bangladesh (47 completions from 123 invitations), Nepal (n = 27) and Nigeria (n = 28); Bangladesh is the principal worked example.

• Combines a common framework with country-specific innovation and scaling content.
• Captures multi-actor profiles, stakeholder positioning, priorities, barriers, and enabling conditions.
• Provides ten reusable digital tools for evidence-informed investment decisions.

## Graphical abstract

**Figure:**
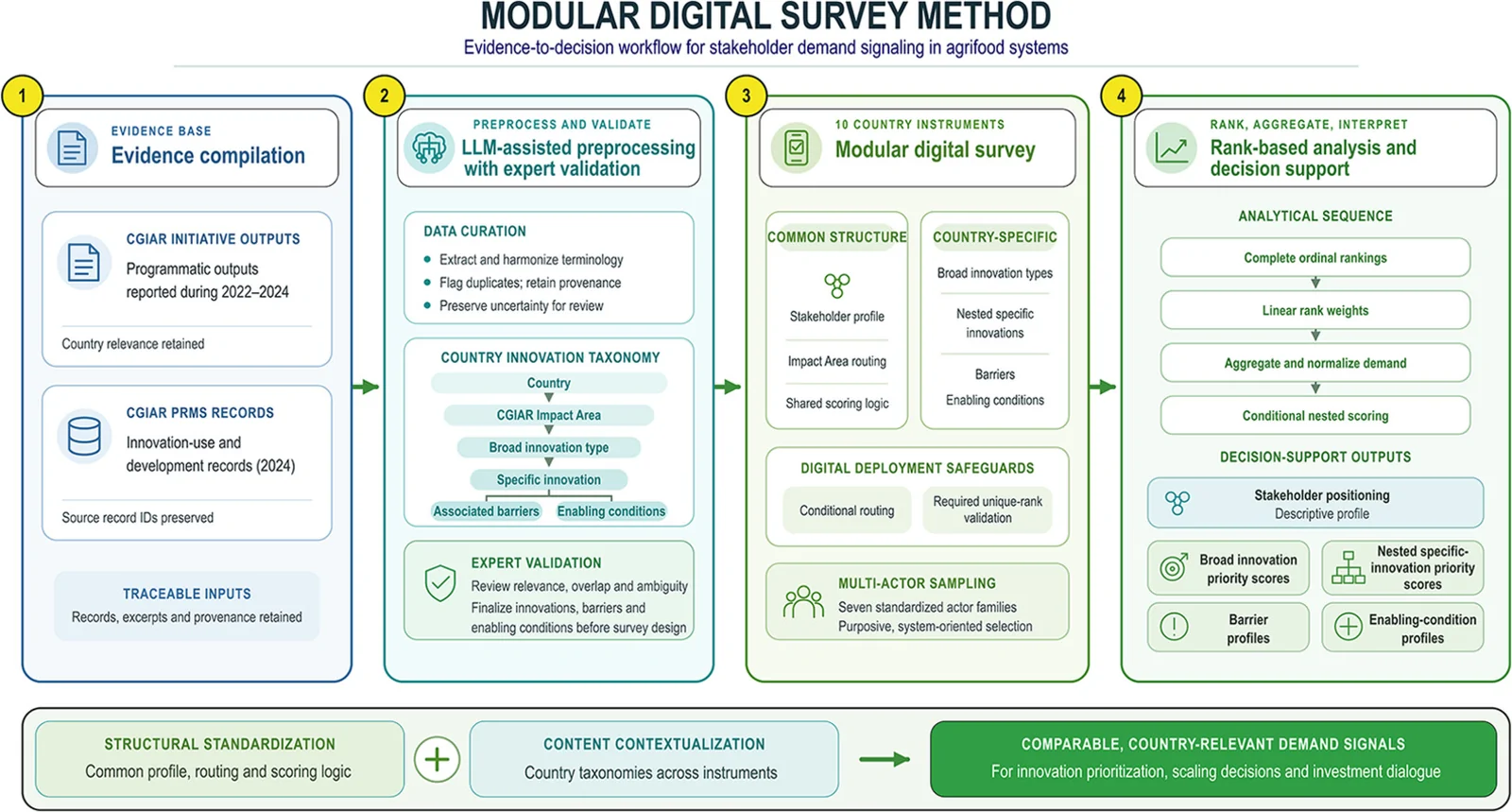

**Specifications table**

**Table:** 

**Subject area**	Agricultural and Biological Sciences
**More specific subject area**	Agricultural innovation systems; stakeholder-informed priority setting; innovation scaling diagnostics
**Name of your method**	Modular multi-actor demand-elicitation and prioritization method for agrifood research and scaling
**Name and reference of original method**	Aravindakshan et al. [1], Stakeholder survey tool on innovation and scaling demands in agrifood systems: A methodology note.
**Resource availability**	Web links to the country-specific instruments, the LLM prompt and output schema, and the scoring equations are included in the manuscript.

## Background

Innovation is not a discrete technical event but an institutional process through which organizations generate, combine, and apply knowledge under historically formed rules and incentives. National and sectoral innovation-system traditions therefore treat learning, interaction, and institutional coordination as constitutive of innovation rather than as conditions external to it [2]. In agrifood systems, this perspective is especially consequential because research organizations, public agencies, firms, intermediaries, civil-society organizations, and producers control different resources required for development, adoption, and scaling [3]. A claim that research is demand-responsive is consequently incomplete unless the relevant actors, their priority ordering, and the institutional conditions attached to those priorities are made observable.

Agricultural Innovation Systems (AIS) and Agricultural Knowledge and Innovation Systems (AKIS) research have shown that demand articulation, intermediation, network coordination, and institutional adaptation influence which innovations are developed and whether they become usable at scale [4,5]. Scaling is therefore not diffusion by multiplication alone: it changes relationships among technologies, users, markets, public authority, and place-specific institutions [[5], [6], [7], [8]]. Priority-setting methods should accordingly elicit both the relative value assigned to innovations and the barriers and enabling conditions actors associate with their use.

Existing exercises often address only part of this problem: innovation inventories describe supply, stakeholder consultations collect heterogeneous narratives, and ranking exercises may suppress the nested relation between broad investment domains and actionable innovations. The method developed here joins these operations in a reproducible sequence. It converts heterogeneous programmatic records into expert-validated country taxonomies before instrument construction; embeds those taxonomies in a common digital architecture; and translates multi-actor rankings into broad, nested, barrier, and enabling-condition scores. Its contribution is methodological rather than empirical. Bangladesh is the principal example of feasibility and output generation; its substantive findings are presented separately [9]. Selected Nepal and Nigeria applications are documented in the supplementary material.

## Method details

### Conceptual framework and design logic

The method begins from a demand-side interpretation of innovation. Linear models represent knowledge as moving from research through development to adoption; innovation-systems scholarship instead locates novelty within recurrent learning among organizations operating under institutional constraints [10,11]. Demand is therefore not inferred from the existence of a technology or from adoption observed after deployment. It is elicited from actors who occupy different positions in the system, while recognizing that preferences are conditioned by mandates, resources, and rules [12].

This conceptual position determines the design logic. Respondent profiling locates each judgment institutionally; hierarchical elicitation separates broad research and investment directions from specific innovations; and linked barrier and enabling-condition modules retain the conditions under which a stated priority could be acted upon [6,5]. The instrument thus treats priority setting as a process of demand articulation rather than a proxy for technical merit, predicted adoption, or social welfare. It also separates two requirements that are often conflated: structural standardization, needed for reproducible administration and analysis, and content contextualization, needed for substantive validity in different country innovation systems [3,13]. Table 1 states the conceptual commitments and their operational consequences.

**Table 1:** Conceptual pillars underpinning the modular digital survey method.

Conceptual pillar	Methodological implication	Operationalization
Innovation as an institutional and interactive process	Demand must be interpreted in relation to actor positions, mandates, and interdependence.	Common profile module and seven-family multi-actor sampling frame.
Demand articulation	Priorities should be elicited rather than inferred from research supply or observed adoption.	Forced ordinal ranking of broad and specific innovations.
Hierarchical priority setting	Strategic domains and actionable innovations must remain analytically connected.	Impact Area routing, broad-type ranking, and nested specific-innovation scoring.
Scaling as system change	Priority signals require information on institutional, informational, financial, infrastructural, and coordination conditions.	Barrier and enabling-condition modules linked to prioritized innovations.
Standardized structure, contextualized content	Cross-country use requires common procedures without imposing identical substantive menus.	Ten instruments share modules, routing, and scoring but use expert-validated country taxonomies.

### Survey development, instrument design and deployment

The method follows an eight-stage sequence (Fig. 1). CGIAR Initiative programmatic outputs (https://www.cgiar.org/research/initiatives-dashboard) and PRMS innovation-use records (https://www.cgiar.org/portfolio-narrative/performance-and-results-management) are first compiled at country level. GPT 5.0-assisted semantic preprocessing then extracts, harmonizes, classifies, and clusters candidate innovations; this step occurs before any survey is written. Experts review the candidate structure and determine the final country taxonomy. Each validated taxonomy is then instantiated in a country instrument that retains a common profile module, routing logic, ranking tasks, and scoring framework. KoboToolbox supports conditional deployment to purposively selected actors, after which rank weighting, aggregation, normalization, and nested scoring generate four core decision-support outputs.

**Fig. 1:**
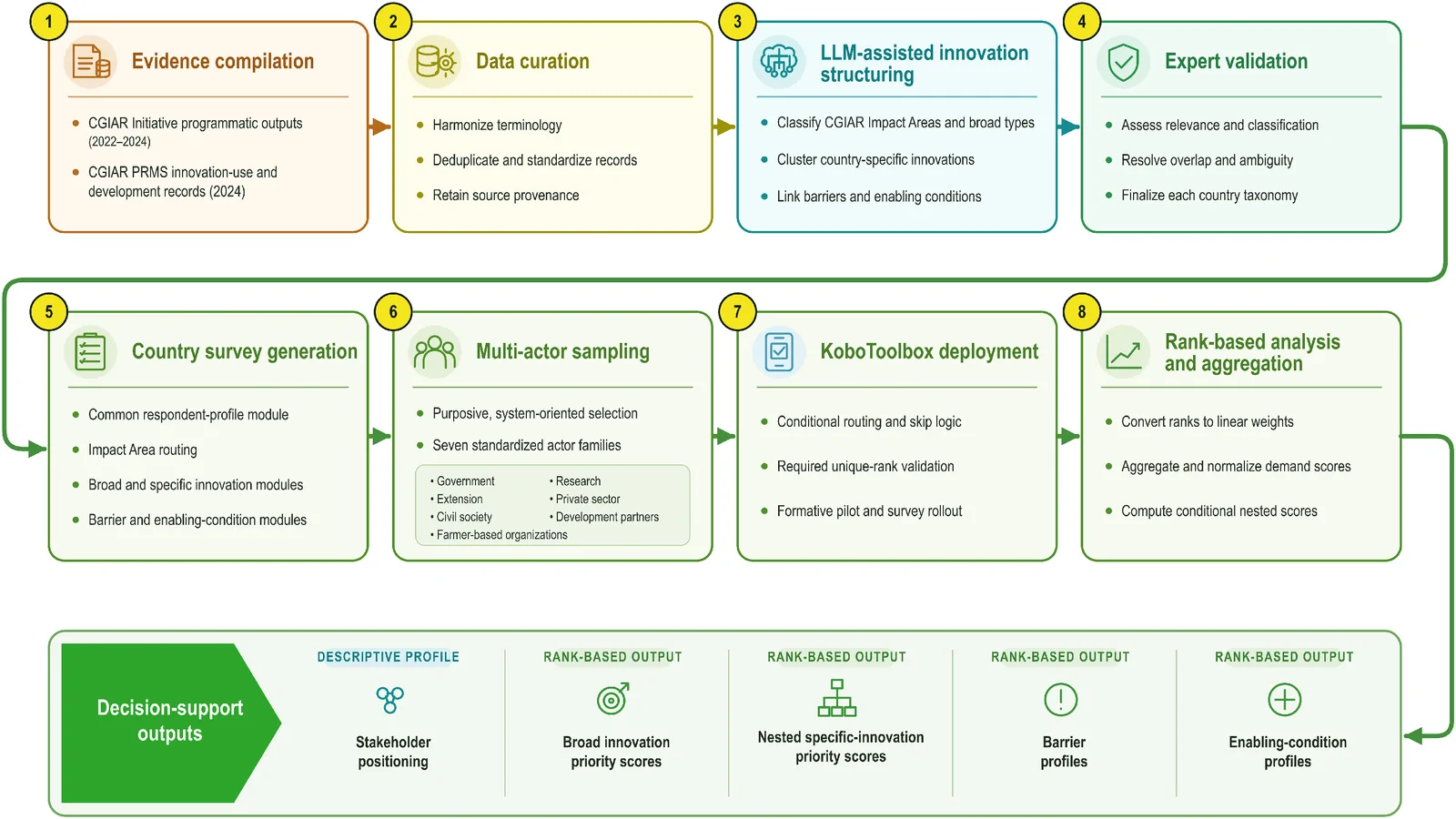
Overall methodological workflow. Programmatic evidence and PRMS records are processed before survey construction; LLM assistance produces candidate structures, while experts determine the final country taxonomy. Ten country instruments share a common architecture and analytical framework but contain country-specific innovation, barrier, and enabling-condition modules.

#### Evidence compilation, LLM-assisted preprocessing, and expert validation

For each country, the evidence base combined 32 CGIAR Initiative programmatic outputs reported during 2022–2024 with 2024 innovation-use and development records from the CGIAR Performance and Results Management System (PRMS) database. Programmatic outputs supplied narrative descriptions, intended users, locations for innovation use, and implementation context; PRMS records supplied structured and semi-structured evidence on innovation identity and readiness. The two sources were treated as inputs into an inventory, not as proof of stakeholder demand. Provenance was retained so that every candidate item could be traced to a source record.

Because the records differed in terminology, granularity, and format, rule-based parsing alone could not reliably identify semantic duplication or consistently separate broad innovation families from specific innovations. GPT 5.0 was therefore used as an assistive large language model (LLM) for semantic extraction, terminology harmonization, preliminary classification, clustering, and organization of candidate barriers and enabling conditions. LLM-supported text classification can accelerate the organization of heterogeneous corpora, but reproducibility depends on constrained prompts, explicit output schemas, retained provenance, and human calibration [[14], [15], [16]]. Model output was consequently treated as a provisional data-curation layer, never as evidence and never as an autonomous inclusion decision.

Processing was conducted through the GPT 5.0 web interface during 2025. The exact model build served by the interface, sampling parameters, access dates for individual runs, and original session logs were not retained; they cannot therefore be reconstructed without qualification. To make the functional procedure auditable, Fig. 2 reports the reusable prompt and output schema reconstructed from the documented workflow. The same task structure and schema were used for all ten countries, with country identifiers and source records varied as inputs. Outputs were mapped to the common sequence country → CGIAR Impact Area → broad innovation type → specific innovation → associated barrier → associated enabling condition, and schema conformity was checked before expert review. The reconstructed prompt documents the intended procedure; it does not reproduce the original sessions.

**Fig. 2:**
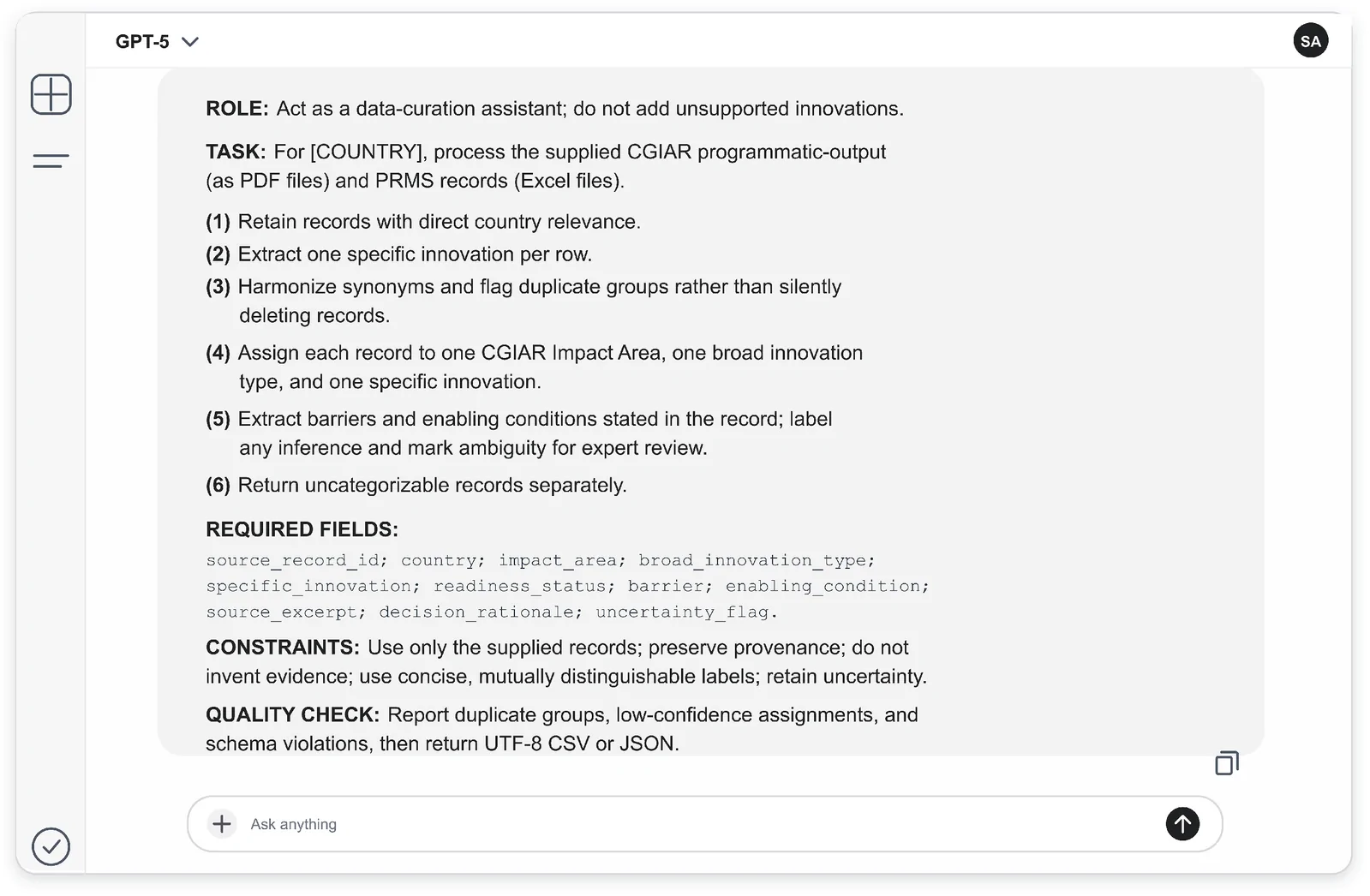
Reconstructed GPT-5 prompt template for preprocessing country-specific CGIAR programmatic outputs and PRMS innovation records.

Two domain specialists reviewed each candidate taxonomy for country relevance, semantic clarity, duplication, category overlap, readiness interpretation, and contextual fit. Flagged records were discussed until a single operational classification was agreed; the reviewers could merge categories, reword labels, reassign items, remove unsupported entries, or return ambiguous records to the source material. The expert-validated taxonomy—not the LLM output—was the input to survey construction (Table 2). Independent agreement statistics and item-level counts of model-generated categories changed during review were not retained, so the procedure supports expert content and face review but not a quantitative claim of classifier accuracy. Table 3

**Table 2:** Inputs and processing steps used to generate country-specific innovation menus.

Input or stage	Period	Material	Function	Auditable output
CGIAR Initiative programmatic outputs	2022–2024	Narrative output records and implementation context	Identify country-relevant candidate innovations and contextual statements	Provenance-linked source inventory
CGIAR PRMS innovation-use data	2024	Structured and semi-structured innovation records	Complement identity and readiness information	Harmonized candidate record set
GPT 5.0-assisted preprocessing	2025	Constrained semantic extraction, classification, and clustering	Propose the hierarchy and linked barriers/enablers; flag ambiguity	Draft schema-conformant country taxonomy
Expert validation	After preprocessing	Two-specialist review and consensus refinement	Determine relevance, wording, category boundaries, and final inclusion	Final country taxonomy used to generate the instrument

**Table 3:** Modules, purposes, data types, and analytical uses in the digital survey instrument.

Module	Purpose	Data	Analytical use
Common respondent profile	Locate each respondent institutionally and by agrifood domain and scaling role.	Categorical and descriptive	Actor- and domain-level disaggregation; interpretation of coverage
Impact Area routing	Direct respondents to the country module matching their primary expertise.	Single selection	Defines the eligible ranking set and structural missingness
Broad innovation types	Elicit relative priorities among country-specific strategic innovation domains.	Complete ordinal ranking	Broad priority score and normalized demand score
Specific innovations	Identify actionable priorities nested within the prioritized broad type.	Conditional complete ordinal ranking	Conditional nested score
Associated barriers	Prioritize constraints linked to the innovations under consideration.	Linked ordinal ranking	Innovation–barrier profile
Associated enabling conditions	Prioritize conditions linked to the innovations under consideration.	Linked ordinal ranking	Innovation–enabler profile

#### Structural standardization and content contextualization

Ten separate country instruments were constructed from the validated taxonomies. Every instrument begins with a common profile module recording institutional affiliation, one of seven harmonized actor families (government, research, extension, private sector, civil society, development partners, and farmer-based organizations), primary agrifood domain of engagement, and role in innovation scaling. These common variables locate judgments within the innovation system and permit transparent disaggregation; they are not used to reweight responses. The remaining content is contextualized: broad innovation types, specific innovations, barriers, and enabling conditions vary with the country evidence base and expert review. A common architecture does not by itself establish numerical equivalence across country-specific menus.

The elicitation sequence moves from coarse to fine (Fig. 3). Respondents select the CGIAR Impact Area closest to their expertise, rank the complete set of country-specific broad innovation types routed to that area, and then rank specific innovations nested within the prioritized broad type. They subsequently rank barriers and enabling conditions linked to the innovations under consideration. Complete rank ordering was selected because it forces explicit trade-offs, records the full ordering rather than only a first choice, and can be implemented transparently in a mobile survey. A simple frequency count would discard information below the top-ranked item. Best–worst scaling can estimate richer preference structures but requires experimentally constructed and repeated choice sets; point allocation elicits intensity but imposes a more demanding cardinal allocation task and can invite heterogeneous scale use [17]. The present method prioritizes low design burden, auditable calculation, and transfer across country-specific menus while acknowledging that its linear weights are scoring conventions rather than utility estimates. Impact Area entry reflects respondents’ selected expertise, not a vote for the area’s investment priority. Nested rankings describe preferences only among respondents routed to the same parent category; differences across branches combine selection and within-menu preference.

**Fig. 3:**
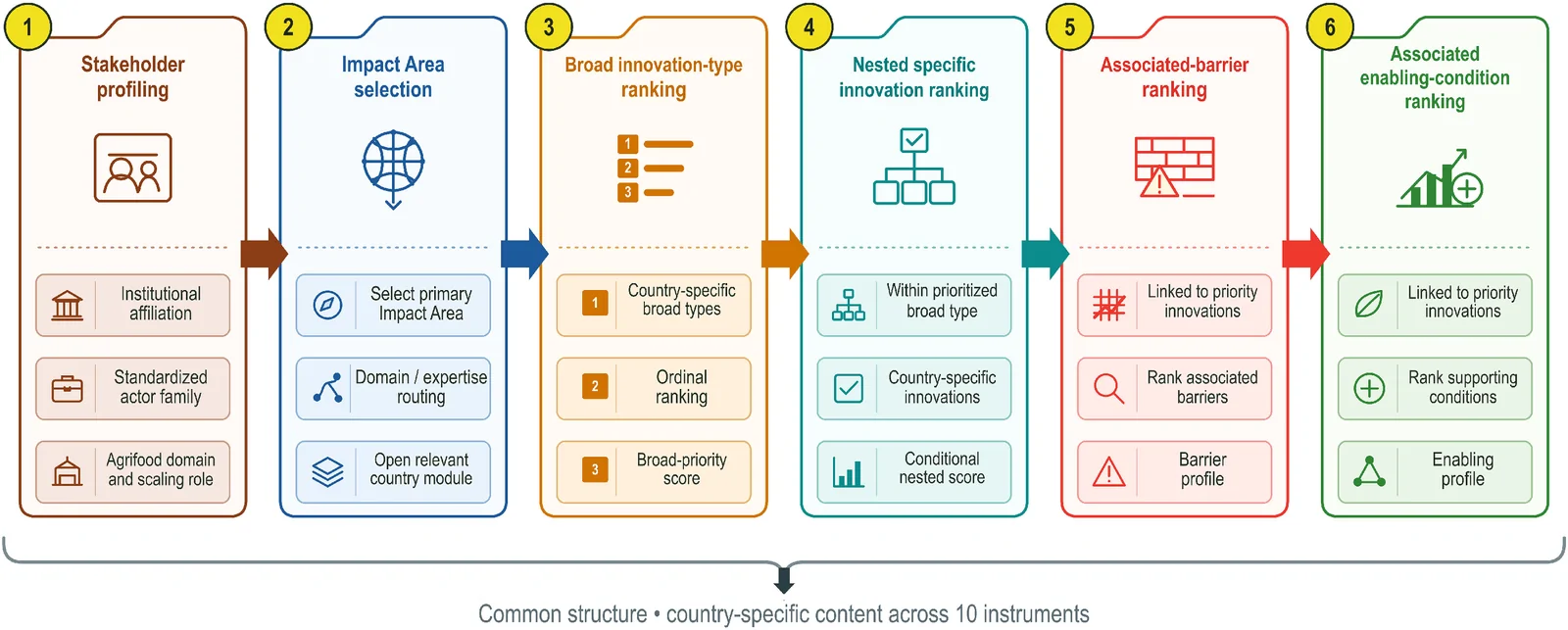
Survey architecture. A common respondent profile and routing sequence are structurally standardized across ten instruments. Impact Areas, broad innovation types, specific innovations, barriers, and enabling conditions are populated from each country’s expert-validated taxonomy. Barrier and enabling-condition modules remain linked to the prioritized innovations.

#### KoboToolbox programming and formative pilot refinement

Each country instrument was programmed in KoboToolbox with relevance conditions, skip logic, required fields, and range and uniqueness constraints. Respondents saw only the modules opened by their Impact Area and broad-type selections. Required complete rankings rejected duplicate or missing rank positions within an eligible set, while non-applicable modules were stored as structurally missing rather than as zero demand. The platform allowed respondents to save an incomplete record and return before submission. Fig. 4 shows the common interface using an illustrative country implementation; the wording and option menus varied by country taxonomy.

**Fig. 4:**
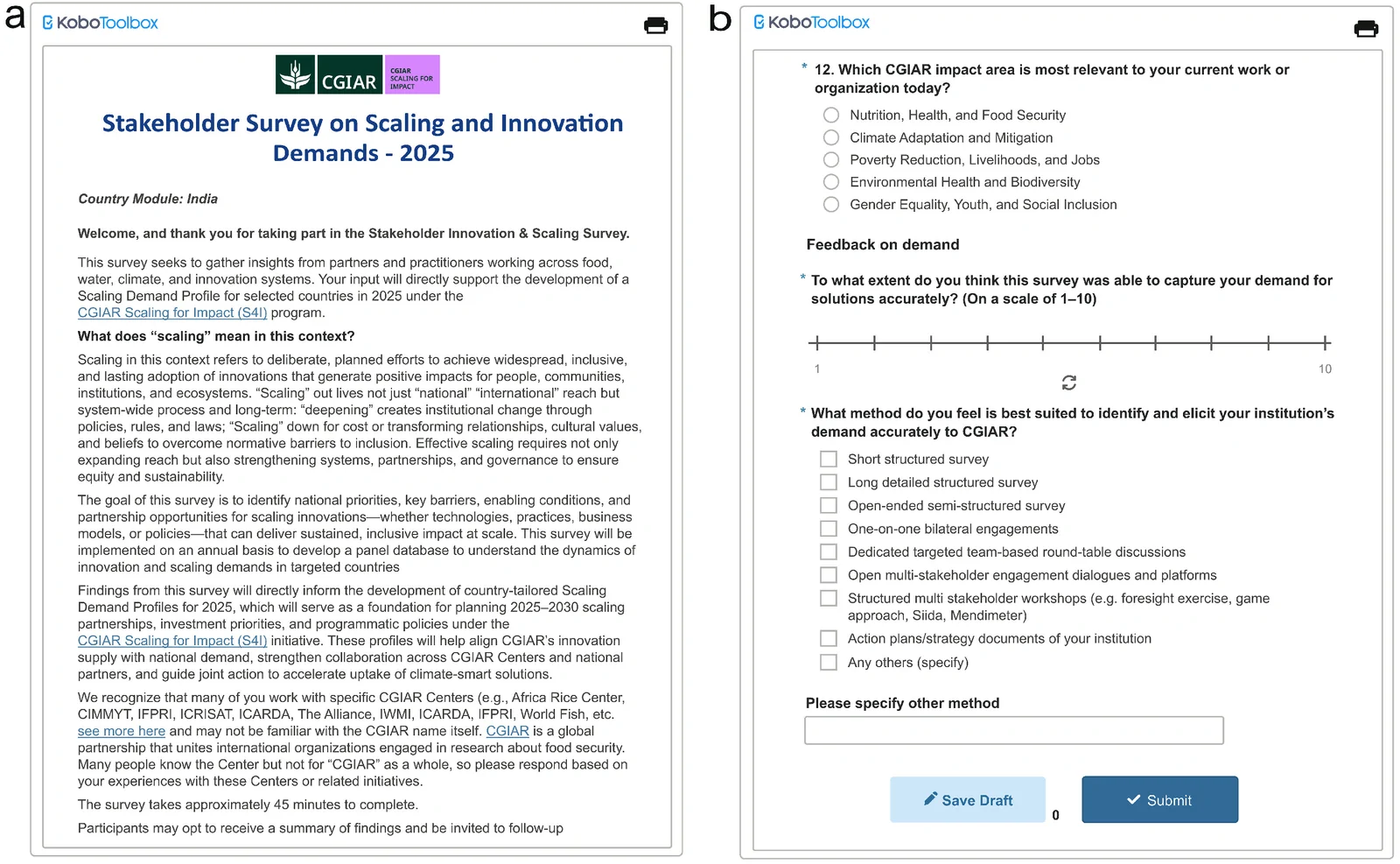
Illustrative KoboToolbox interface from one country instrument. The profile layout, navigation, Save Draft function, submission logic, and validation structure were common across instruments; country names and substantive option menus varied with the validated taxonomy.

A formative usability pilot with five respondents was used to detect comprehension problems, routing failures, excessive ranking burden, and unclear terminology; it was not designed for statistical or psychometric validation. The initial version required approximately 70 min on average, a duration likely to amplify fatigue and satisficing in repeated cognitive tasks [18]. The team reduced broad-option sets to fewer than 12 items, shortened descriptions, removed avoidable acronyms, substituted trait-based descriptions for varietal or breed names, and clarified the partner-ranking question. The revised instrument required approximately 40 min in subsequent use. These approximate observations informed usability refinement; they do not estimate a field-wide completion-time distribution or a controlled reduction in operational evidence of improved usability, not evidence of construct validity [19]. The ten country-specific digital survey instruments developed through the eight-step process illustrated in Fig. 1 are presented in Table 4. Field applications are documented for Bangladesh, Nepal and Nigeria; instruments for the other seven countries were prepared but not field-deployed.

**Table 4:** Country-specific digital survey instruments by CGIAR region.

Country	CGIAR region	Digital survey instrument
Egypt	Central and West Asia and North Africa (CWANA)	https://ee-eu.kobotoolbox.org/x/dN9bKxvK
Ethiopia	East and Southern Africa (ESA)	https://ee-eu.kobotoolbox.org/x/mBqi2Ym1
Kenya	East and Southern Africa (ESA)	https://ee-eu.kobotoolbox.org/x/4I91HX6E
Nigeria	West and Central Africa (WCA)	https://ee-eu.kobotoolbox.org/x/8QoV5Avh
Bangladesh	South Asia (SA)	https://ee-eu.kobotoolbox.org/x/BMcCB9ZN
India	South Asia (SA)	https://ee-eu.kobotoolbox.org/x/trO0Iyvm
Nepal	South Asia (SA)	https://ee-eu.kobotoolbox.org/x/1HTTFgby
Vietnam	Southeast Asia and the Pacific (SEA)	https://ee-eu.kobotoolbox.org/x/qBPZ4A3q
Guatemala	Latin America and the Caribbean (LAC)	https://ee-eu.kobotoolbox.org/x/jIaQHLq0
Colombia	Latin America and the Caribbean (LAC)	https://ee-eu.kobotoolbox.org/x/EsvLXuFk

Note: All ten country-specific instruments were programmed using KoboToolbox. Field applications are documented for Bangladesh, Nepal and Nigeria; the other seven instruments were prepared but not field-deployed. Regional groupings follow CGIAR’s six-region classification (https://www.cgiar.org/cgiar-regions). The links document the country-specific instrument versions and do not indicate current survey availability.

### Sampling strategy and respondent selection

Sampling is purposive and system-oriented. The target population is not a statistically homogeneous public but the set of organizations whose authority, knowledge, finance, delivery functions, market position, or collective action bears on innovation and scaling. Country teams first identify organizations in each actor family and then nominate knowledgeable respondents occupying roles relevant to research, implementation, financing, regulation, commercialization, or user representation. The objective is analytical coverage of functions and perspectives, not population prevalence or design-based generalization [20].

The common frame comprises seven actor families: government; research; extension; private sector; civil society; development partners; and farmer-based organizations. These families correspond to distinct but overlapping functions in an innovation system and provide a harmonized level for comparing participation across country instruments. Country-specific organizational labels may be retained during recruitment and reporting, but they are mapped to this common frame before analysis. Sampling matrices should record invitations, completions, actor-family coverage, and obvious gaps so that priority scores can be interpreted in relation to who participated rather than presented as population estimates [3,4]. In Bangladesh, 47 of 123 invitations yielded completed questionnaires (38.2%); 76 did not yield a completion. Reasons for noncompletion and matched invitation/completion counts by actor family are not available in the reported summaries, so differential nonresponse cannot be assessed. Fig. 5 describes achieved institutional participation, and Table 6 reports Impact Area entry counts. Supplementary Table S1 provides the corresponding Nepal and Nigeria composition and entry counts. These purposive samples do not support population inference.

**Fig. 5:**
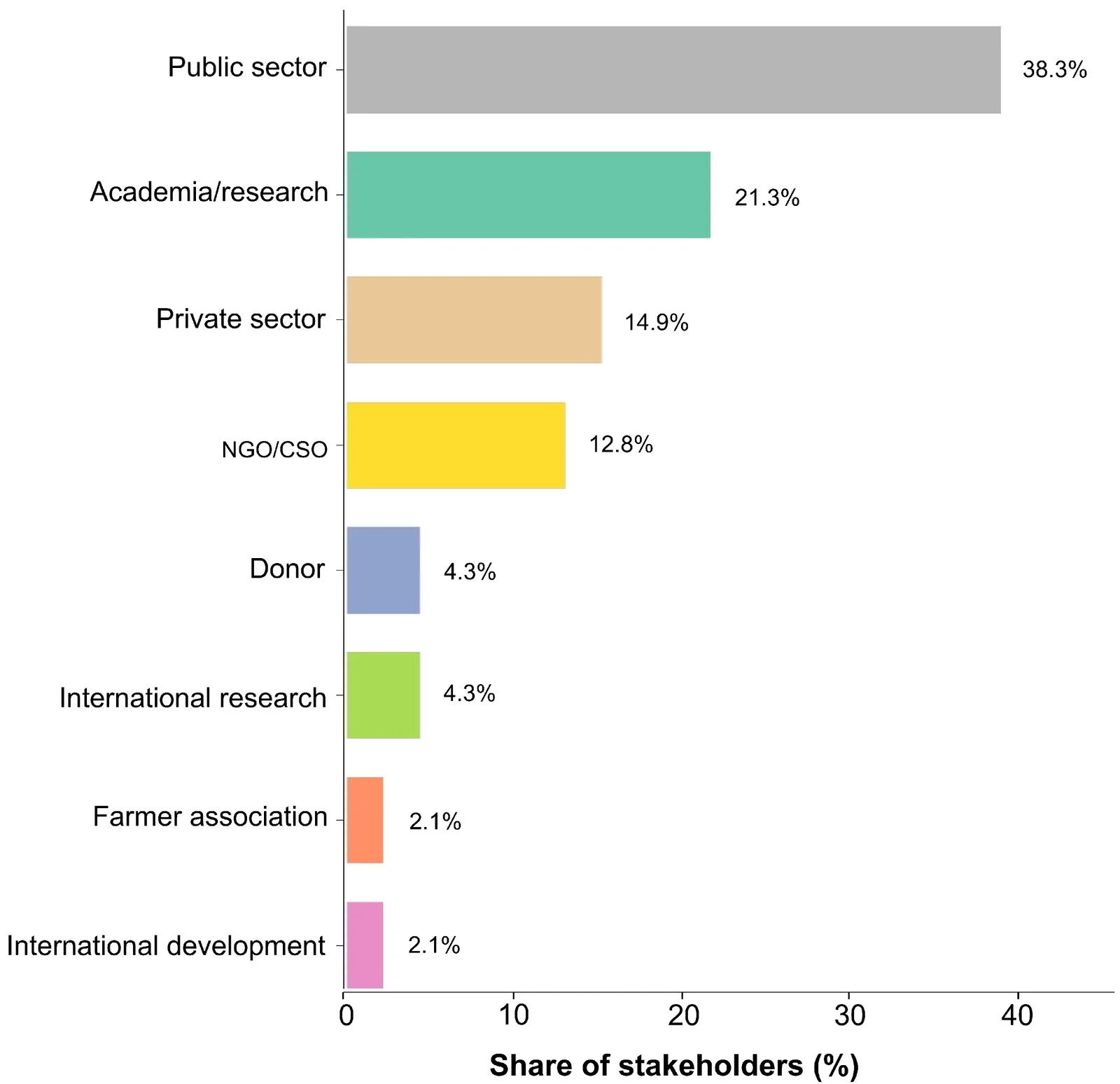
Respondent composition in the Bangladesh feasibility application (n = 47). Country-specific institutional labels are retained in the chart and can be mapped to the seven common actor families for cross-country coverage assessment. This is a sampling output, not a demand score.

### Data processing and analytical framework

Submitted records are first checked for consent, completion status, routing consistency, duplicate identifiers, and valid rank permutations. Analysis is restricted to respondents eligible for a module; structural missingness created by routing is not recoded as a zero. Within a required ranking set containing Rmax items, each respondent supplies a unique permutation from 1 (highest priority) to Rmax (lowest priority). The method then converts rank positions to linear weights, aggregates those weights among eligible respondents, normalizes within the relevant option set, and preserves the hierarchy between broad and specific innovations [21]. The same sequence is applied separately to innovation, barrier, and enabling-condition rankings.

Let $r_{ij}$denote the rank assigned by respondent ito item j, where lower numerical ranks indicate higher priority, and let $R_{\text{max}}$denote the number of items, and therefore the maximum rank, in that respondent’s eligible module. The rank-derived weight assigned to item jby respondent iis defined as:

(1) $$\begin{array}{cccc} & w_{ij}=\left(R_{\text{max}}+1\right)-r_{ij} & & \end{array}$$

Eq. (1) assigns Rmax points to a first-ranked item and one point to the last-ranked item. It preserves the complete ordering and gives every rank position analytical influence. The equal decrement between adjacent ranks is an explicit Borda-type scoring assumption; it does not imply that respondents experience equal psychological distances between successive preferences.

The aggregate score for item jis calculated as:

(2) $$\begin{array}{cccc} & S_{j}=\sum_{i=1}^{N}w_{ij} & & \end{array}$$

where N is the number of respondents evaluating item j. Within a required complete ranking, the same eligible respondents evaluate all items, so *N_j_* is common and $S_{j}$ represents collective rank-weighted priority. If item exposure or completion differs, raw sums also reflect denominator size; analysts should then restrict comparison to a common eligible set or report the mean weight *S_j_/N_j_* alongside S*_j_*.

To facilitate comparison across items within a module, the aggregate scores are normalized as:

(3) $$\begin{array}{cccc} & ND_{j}=\frac{S_{j}}{\text{max}\left(S_{j}\right)}\times \;100 & & \end{array}$$

where $ND_{j}$ is the normalized demand score on a 0–100 scale. The maximum item in the analytic set is therefore anchored at 100 and all other values are relative to it. $ND_{j}$ supports comparison within the same module and respondent set; it is not an absolute demand scale and should not be compared across differently worded country menus or routing structures without demonstrating measurement equivalence. Normalization does not correct route selection or make scores comparable across modules, parent categories or country menus. A score of 100 denotes only the maximum within its specified respondent set and option menu.

Because the survey is hierarchical, the analytical framework also computes conditional scores for specific innovations nested within broad innovation categories:

(4) $$\begin{array}{cccc} & S_{k|j}=\sum_{i=1}^{N_{j}}w_{ik|j} & & \end{array}$$

where $N_{j}$is the number of respondents who evaluated specific innovation set j, and $w_{ik|j}$is the weight assigned to specific innovation kunder broad category j. This nested formulation allows the method to identify both high-level innovation and scaling demand domains and finer-grained priorities within them. Parent selection and conditional child rankings should be reported separately, with the eligible count for each parent-specific menu. Impact Area entry counts are not automatically the denominators for nested rankings.

Eqs. (1)–(3) are applied independently to broad innovation types, linked barriers, and linked enabling conditions; Eq. (4) retains the conditional relation between a specific innovation and its parent category. For each output, analysts should report the eligible denominator, option menu, normalization set, the raw aggregate score, and normalized score [22]. Actor-family or agrifood-domain disaggregation is appropriate only when subgroup cell sizes and option exposure are reported. Sensitivity checks may recalculate results using top-choice frequency, mean rank, or alternative monotonic weights; convergence strengthens confidence in the ordering, whereas divergence should be presented as dependence on the scoring rule. Table 5 defines the notation and the boundaries of interpretation.

**Table 5:** Analytical notation and interpretation of scoring equations.

Symbol	Definition	Interpretation
$r_{ij}$	Rank assigned by respondent *i* to item *j*	1 is highest priority; rankings form a unique complete order within the eligible set
$R_{\text{max}}$	Number of items and maximum rank in the eligible module	Defines the ranking scale within the eligible option menu
$w_{ij}$	Linear rank weight assigned by respondent *i* to item *j*	*Rmax* for first place and 1 for last place; not a cardinal utility
$S_{j}$	Aggregate rank-weighted score for item *j* among *N_j_* eligible respondents	Collective priority only for a common respondent set and option menu; report *N_j_*
$ND_{j}$	Normalized demand score for item *j*	Maximum of 100 within the specified respondent set and menu; no cross-route scale
$S_{k|j}$	Conditional aggregate score for specific innovation *k* within broad category *j*	Priority conditional on parent selection; report the parent-specific eligible count

### Output generation and empirical application

The analytical framework produces four core ranked outputs: broad innovation priorities, conditional priorities for specific innovations, barrier profiles, and enabling-condition profiles. Profile variables add descriptive information on the composition and institutional positioning of participants but are not a fifth demand score.

Bangladesh is the principal worked example of feasibility and output generation. Forty-seven of 123 invited actors submitted complete questionnaires (38.2%). Fig. 5, Fig. 6, Fig. 7, Fig. 8, Fig. 9, Fig. 10 are deliberately selective illustrations of output form: respondent coverage (Fig. 5), cross-cutting domain scores (Fig. 6), descriptive stakeholder positioning (Fig. 7), one nested thematic module (Fig. 8), and linked barrier and enabling-condition profiles (Fig. 9, Fig. 10). They are not presented as the empirical contribution of this paper; the country-specific interpretation and full results appear in Aravindakshan et al. [9].

**Fig. 6:**
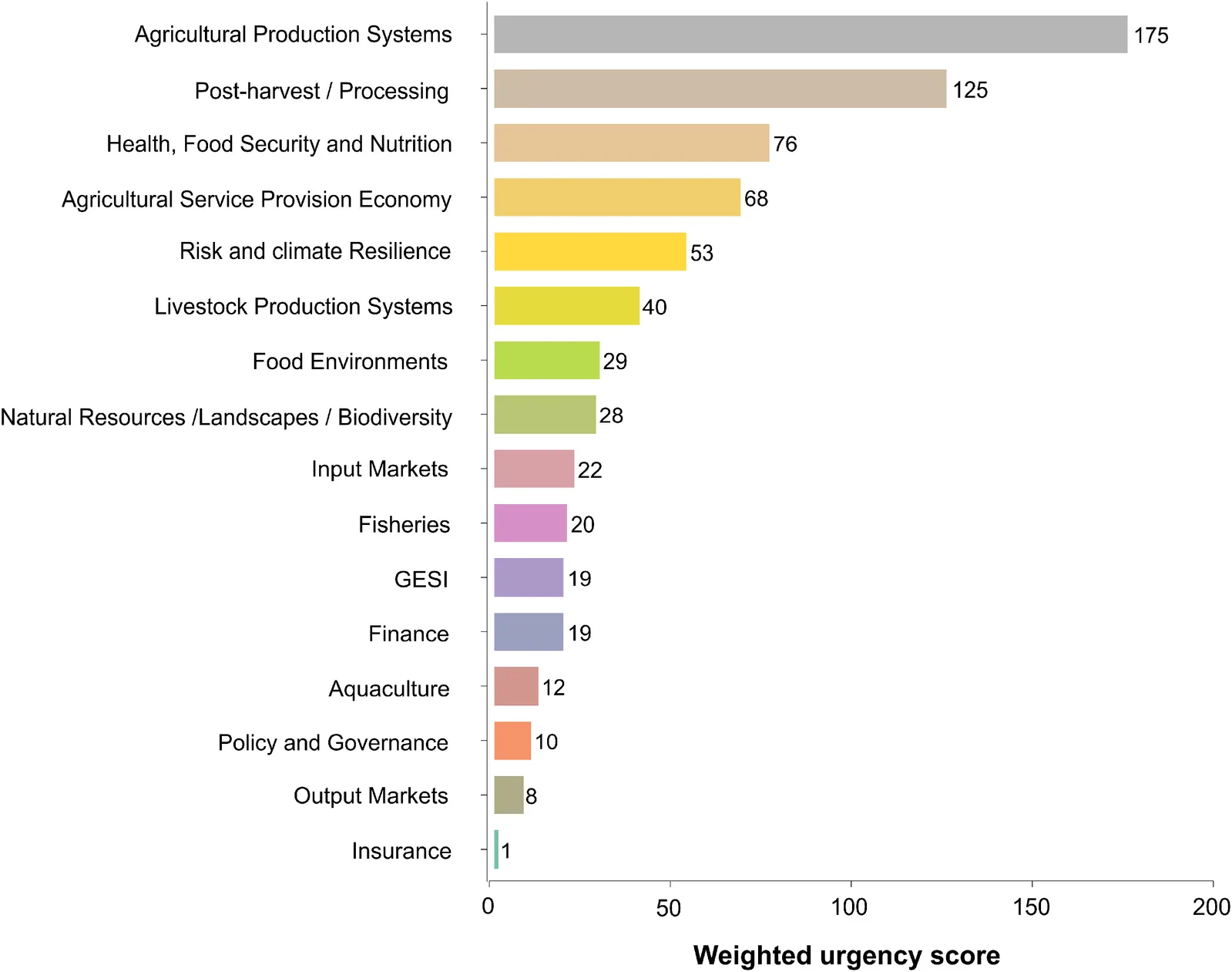
Illustrative cross-cutting domain output from the Bangladesh application (country n = 47). Bars reproduce the reported weighted urgency totals; they are not normalized demand indices or population estimates. This ranking task is separate from Impact Area routing. A valid-case count for this specific task is not separately reported in the application summary.

**Fig. 7:**
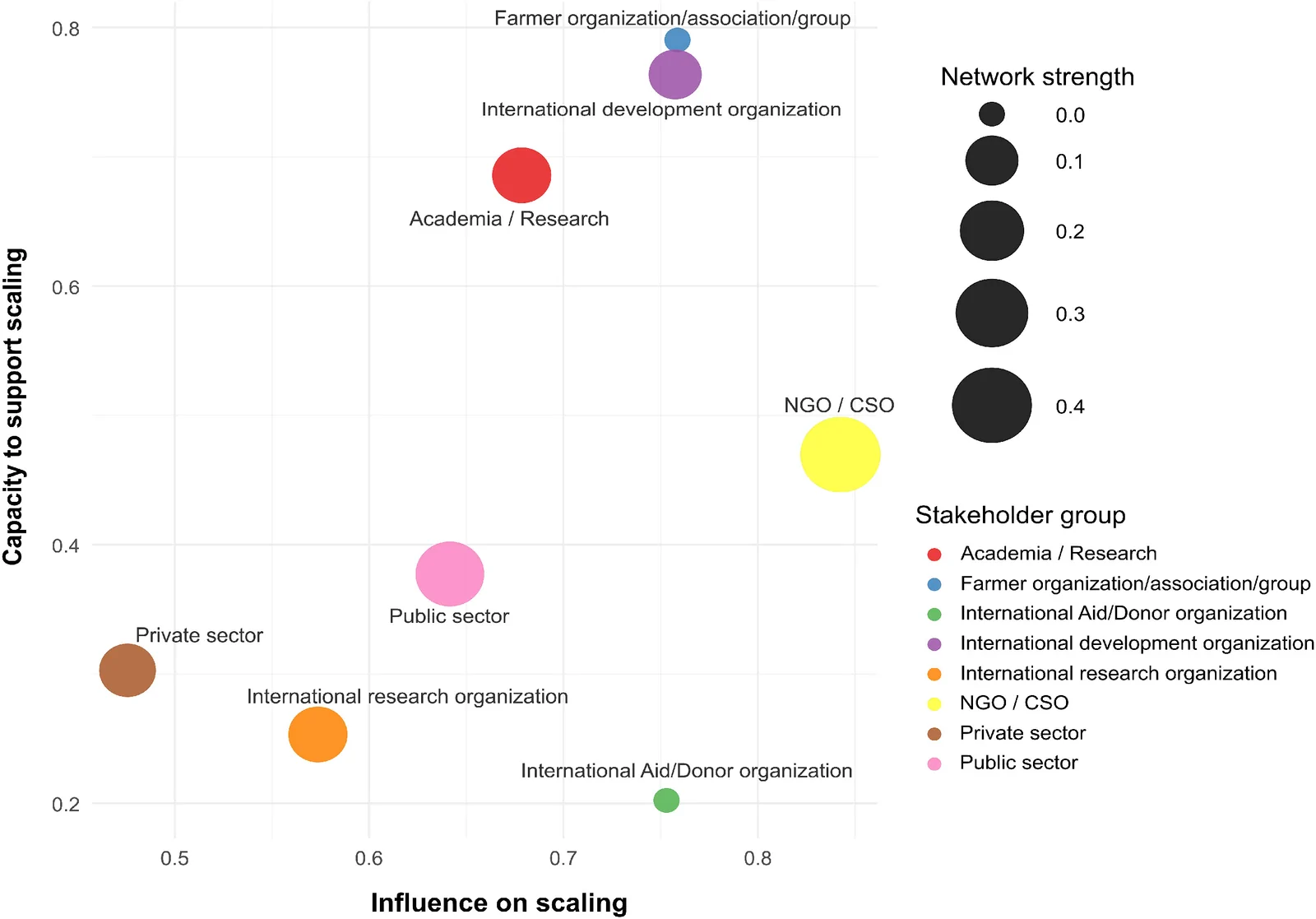
Illustrative stakeholder-positioning output from the Bangladesh profile module. Position summarizes self-reported influence and capacity, while bubble size represents network strength. This descriptive segmentation supports interpretation but is not one of the four core rank-weighted demand outputs.

**Fig. 8:**
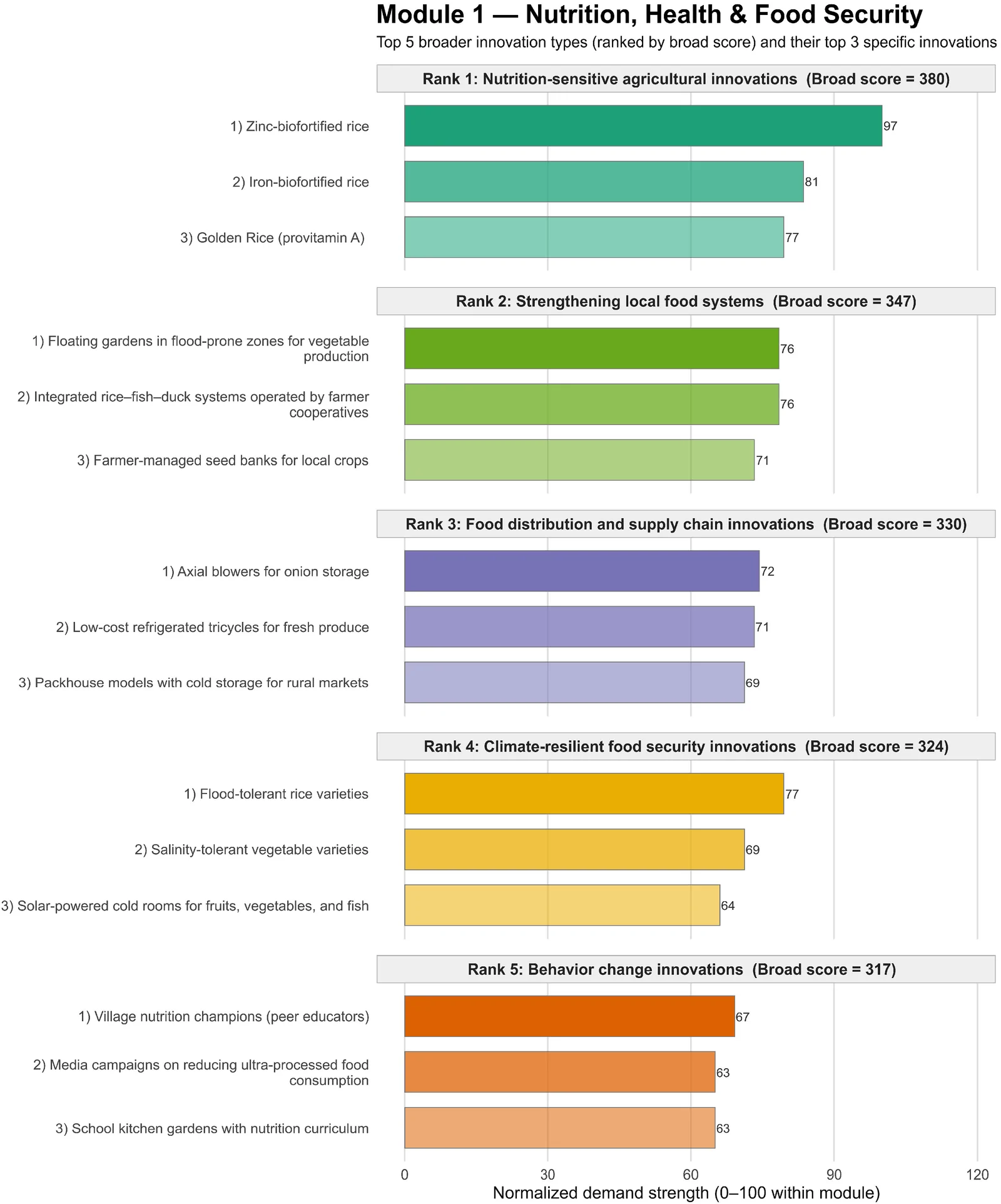
Illustrative hierarchical output for the Nutrition, Health and Food Security module (route) in Bangladesh (Impact Area entry n = 21). Bars show normalized demand scores on a 0–100 scale for selected specific innovations; the panel headings report separate broad-category scores. *Child*-item comparisons are restricted to the same *parent*-specific menu and eligible respondent set. Parent-specific valid-case counts are not reported in the application summary; the entry count is not a substitute for those denominators.

**Fig. 9:**
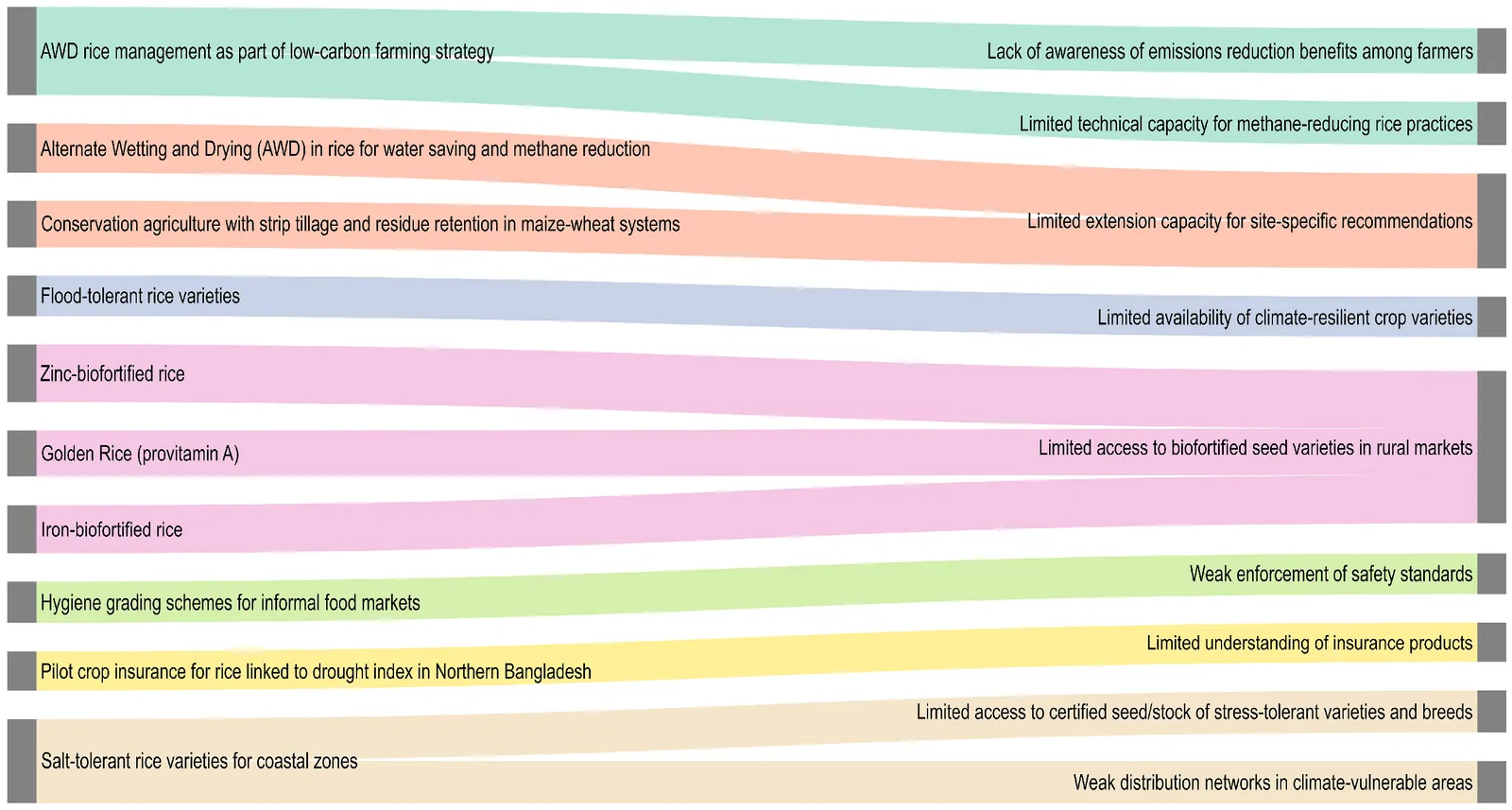
Illustrative innovation–barrier profile from Bangladesh. Flow widths show the relative prominence of barriers linked by respondents to selected innovations; the diagram preserves the association rather than reporting barriers as context-free frequencies. Links arise from respondents’ selected routes; widths across different routes do not define a common priority scale. Link-specific eligible counts are not reported in the application summary.

**Fig. 10:**
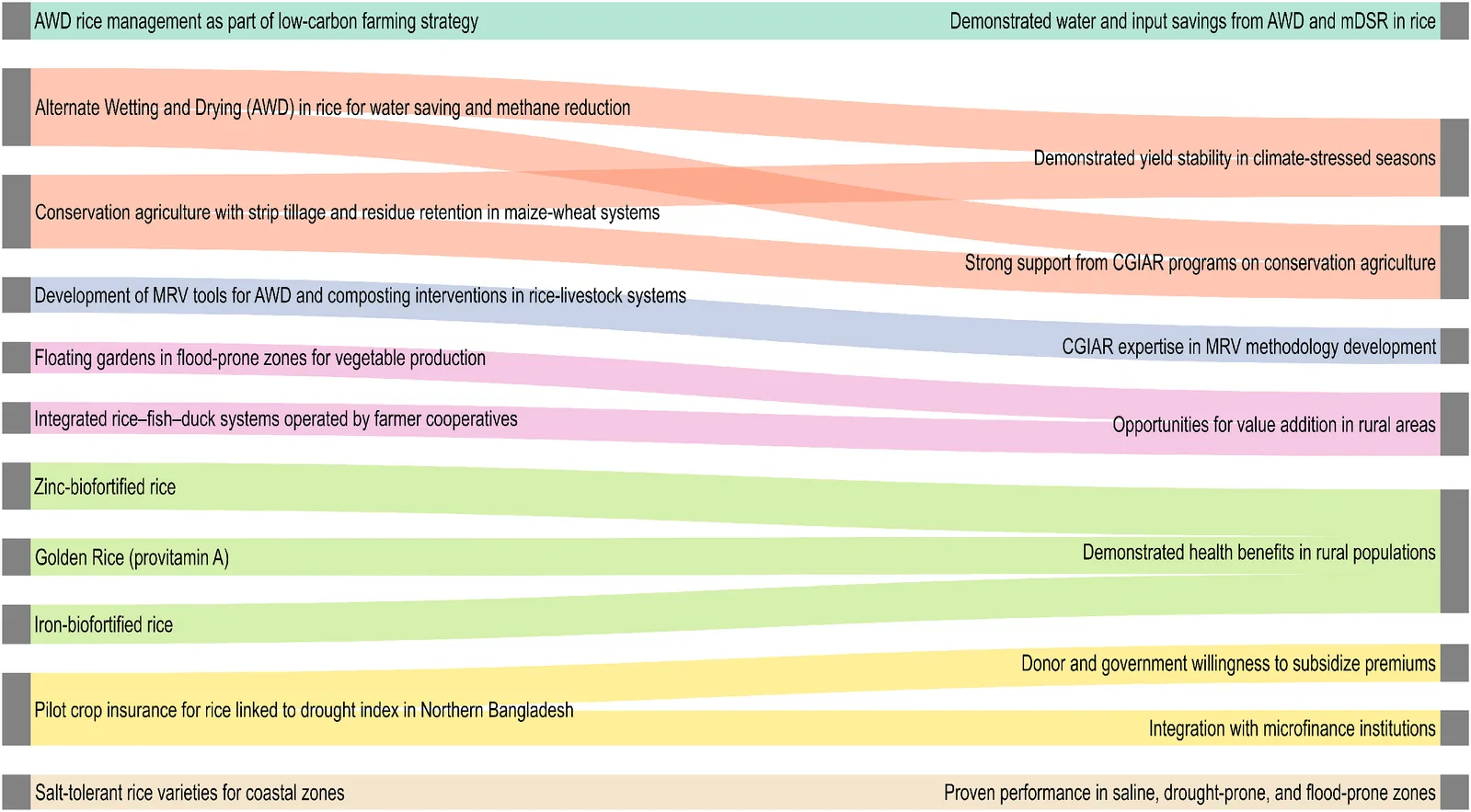
Illustrative innovation–enabling-condition profile from Bangladesh. Flow widths show the relative prominence of conditions linked to selected innovations, complementing the barrier profile in Fig. 9. Comparisons require the same route, option menu and eligible respondent set. Link-specific eligible counts are not reported in the application summary.

Table 6 shows that Bangladesh participation was concentrated in Nutrition, Health and Food Security and Climate Adaptation and Mitigation (21 respondents each); the other three Impact Areas had one to three respondents. These are entry counts, not a common scale of demand or the denominators for every nested ranking. Nepal (n = 27) and Nigeria (n = 28) provide additional applications in Supplementary Table S1 and Figures S1–S2, with operational summaries in Table S2. Country samples remain separate, and raw scores are not interpreted as cross-country differences in demand.

**Table 6:** Bangladesh participation by CGIAR Impact Area.

Completed questionnaires / Impact Area	Bangladesh
All completed questionnaires	47
Nutrition, Health and Food Security	21
Climate Adaptation and Mitigation	21
Poverty Reduction, Livelihoods and Jobs	3
Environmental Health and Biodiversity	1
Gender Equality, Youth and Social Inclusion	1

Note: Counts refer to the selected Impact Area in Question 12 and sum to 47. Small cells are descriptive and should not be used for subgroup inference. Entry counts do not establish valid-case counts for nested rankings.

### Ethical and operational considerations

Participation is voluntary and begins with digital informed consent. Respondents may decline participation, omit non-required profile items, save an incomplete record, or withdraw before submission. Identifiers are removed before analysis and access to raw responses is restricted. Because profile combinations can make individuals recognizable in small professional communities, subgroup reporting should suppress sparse cells. The instrument is intended for strategic learning and priority setting, not for evaluating individual performance. CIMMYT’s Institutional Research Ethics Committee approved the tool and application (IREC 2025.028).

## Method validation

Validation focused on content relevance, procedural coherence, expert verification of country-specific taxonomies, and the integrity and interpretability of the survey’s routing, ranking, and scoring outputs. Psychometric construct validity was not assessed because the instrument does not estimate a latent construct from correlated multi-item measures; it directly elicits stakeholder characteristics and context-specific ordinal judgments concerning innovation priorities, barriers, and enabling conditions. Its conceptual validity is therefore supported by the alignment of actor profiling, hierarchical demand elicitation, and scaling diagnostics with established innovation-systems and scaling theory [3,5,6]. Content and face review occurred when two domain specialists examined the country taxonomies and questionnaire wording. These steps support relevance and interpretability, but they do not establish construct validity, criterion validity, or reliability.

Procedural validation combined a five-person formative usability pilot with field application in Bangladesh. The approximate 70-minute initial-pilot and 40-minute subsequent-use observations informed refinement but do not establish a controlled reduction in completion time. Bangladesh yielded 47 completed questionnaires from 123 invitations and generated the four pre-specified ranked outputs plus profile visualizations [9]. Selected Nepal and Nigeria outputs extend the record of field application; they do not establish validation in all ten countries. Table 7 separates the validation claims, while Table 8 reports the Bangladesh operational measures.

**Table 7:** Validation dimensions and evidence supporting the modular digital survey method.

Validation dimension	Assessment	Evidence	Permitted inference
Conceptual coherence	Map modules and outputs to innovation-systems, demand, and scaling theory	Actor profile, nested priorities, and linked scaling diagnostics form a consistent design	The method operationalizes its stated conceptual framework
Content and face validity	Two-specialist review and consensus refinement of country taxonomies and wording	Final country-specific menus judged relevant and understandable	Items are contextually defensible; classifier accuracy is not estimated
Formative usability	Five-person pretest focused on comprehension, routing, and burden	Approximate observations of 70 min initially and 40 min in subsequent use; wording and option sets revised	Formative usability evidence; no controlled estimate of burden reduction or psychometric inference
Analytical reproducibility	Recalculate rank weights, aggregate scores, normalization, and nested scores from exports	Prespecified equations generated traceable outputs	The transformation from ranks to reported scores is auditable
Empirical feasibility	Bangladesh worked example; selected Nepal and Nigeria applications	Bangladesh: 47/123 completions; Nepal: 27; Nigeria: 28; operational summaries in Table 8 and Table S2	Evidence of implementation in three settings; not ten-country validation or population representativeness

**Table 8:** Reported operational measures for the Bangladesh application.

Measure	Bangladesh
Submitted records	47
Reported routing inconsistencies	0/47 (0%)
Submissions without informed consent	0/47 (0%)
Invalid ranking options available in the tool	None
Reported missing or lost submission records	0
Reported device/power interruptions	1/47 (2.1%); resumed after charging
Records retained for elapsed-time analysis	47
Median elapsed completion time, minutes	53.7
Interquartile range, minutes	32.1–85.9
Tool design and testing: estimated average effort per survey	5 person-days; 40 h
Data curation and analysis: reported effort per country	4 person-days; 32 h
Combined estimated effort for these activities	9 person-days; 72 h
Broad-option menu length after pilot refinement	Fewer than 12 items; visited-route distribution not reported
Eligible-item completion and invalid saved rankings	Not quantified in the reported summary
Rejected submission attempts and corrected/excluded records	Not quantified in the reported summary
Ranking tasks per route and parent/link-specific valid-case counts	Not quantified in the reported summary

Note: Values are reported operational summaries, not an independent reanalysis of raw submissions or process logs. Zero reported events are limited to the specified measures and submitted-record base; they do not establish the absence of unrecorded pre-submission failures. “None” for invalid options describes tool design, not a measured invalid-submission rate. Elapsed times may include interruptions. Effort totals imply an eight-hour person-day and cover only the activities listed.

For Bangladesh, the reported summary records no routing inconsistencies or submissions without informed consent among 47 submitted records, no reported missing or lost submissions, and one device/power interruption after which the survey resumed. Median elapsed completion time was 53.7 min (IQR 32.1–85.9; n = 47). Elapsed time may include pauses or save-and-return intervals and is not equivalent to active interview time. Tool design and testing were estimated at five person-days (40 h), with four person-days (32 h) reported for data curation and analysis. These figures describe the stated activities; they are not a complete survey budget or a time-and-motion assessment.

Required fields and ranking constraints are design safeguards, not measured item-level completion rates. The reported summaries do not quantify rejected attempts, invalid saved rankings, eligible-item missingness, corrected or excluded records, or the distribution of menu lengths and ranking tasks across visited routes. These measures are not assigned zero values. Parent-specific and link-specific valid-case counts also remain unavailable for the reproduced country illustrations.

## Limitations

The method is designed for analytically targeted demand signaling rather than population estimation or causal attribution. Purposive, network-informed recruitment captures perspectives across relevant actor families, while subsequent applications can extend coverage through targeted inclusion of informal or less-connected groups. Rankings represent perceived priorities—the intended decision signal—rather than causal effects on adoption, welfare, or sustainability. Linear rank weighting provides a simple and transparent basis for aggregation but does not measure preference intensity, making modular routing and manageable option sets important design safeguards. Country-specific content strengthens contextual relevance. However, Impact Area and parent-category self-selection condition the respondent base, and normalization does not remove this selection. Scores therefore do not provide a common priority scale across routed modules, parent categories or country menus. Expert review and formative piloting assessed content relevance, clarity, routing, and usability, an appropriate validation strategy because the instrument elicits actor characteristics and ordinal judgments rather than latent psychometric constructs. Source provenance, a reconstructed prompt and expert review document the intended preprocessing workflow, but the original GPT sessions cannot be replayed exactly because model identifiers, settings and session logs were not retained. Ding and Wang [23] emphasize verifiable environments for reliable agentic execution. In this narrow auditability context, future preprocessing should retain versioned inputs, model/interface identifiers, prompts, outputs and expert-edit records; this recommendation does not validate the agrifood framework or reconstruct the original sessions. Matched actor-family recruitment records, actor-by-module cell sizes, eligible-item missingness and parent-specific valid-case counts are not available in the reported summaries, limiting assessment of nonresponse and conditional outputs.

## Ethics statements

The study involved human participants. Digital informed consent was obtained before survey participation. Participation was voluntary, respondents could withdraw before submission, and data were anonymized before analysis. The survey instrument and its application were approved by CIMMYT’s Institutional Research Ethics Committee (IREC 2025.028).

## CRediT author statement

**Sreejith Aravindakshan:** Conceptualization, Methodology, Software, Formal analysis, Visualization, Writing – original draft, Writing – review and editing. **Timothy J. Krupnik:** Conceptualization, Methodology, Supervision, Writing – review and editing, Funding acquisition. **Dyutiman Choudhary:** Writing – review and editing. **Shreya Chakraborty:** Writing – review and editing. **Mustafa Kamal:** Software, Validation, Data curation, Writing – review and editing. **Md. Harun-Or-Rashid:** Data curation, Validation, Writing – review and editing. **Gerald Atampugre:** Writing – review and editing. **Olufunke Cofie:** Writing – review and editing. **Paswel Marenya:** Writing – review and editing**. Deissy Martínez-Barón:** Writing – review and editing.

## Declaration of competing interest

The authors declare that they have no known competing financial interests or personal relationships that could have appeared to influence the work reported in this paper.

## Data Availability

Data will be made available on request.
