# Supplementary material for: A modular digital method for eliciting multi-actor demand to prioritize agrifood research and scaling investments
Source: MethodsX. 2026 Oct 19;17:104220. doi: 10.1016/j.mex.2026.104220 (PMC13735001; doi:10.1016/j.mex.2026.104220)
Supplement: Supplementary file 1 [file mmc1.docx]

Supplementary material

**(Additional field applications in Nepal and Nigeria)**

**A modular digital method for eliciting multi-actor demand to prioritize agrifood research and scaling investments**

Nepal and Nigeria contributed 27 and 28 completed questionnaires, respectively, from purposively recruited stakeholders [1, 2]. Their decision-making roles give the elicited priorities practical relevance but do not remove selection bias. Table S1 describes achieved participation, Figures S1–S2 illustrate cross-cutting outputs, and Table S2 reports operational measures. Country samples remain separate; these applications do not constitute ten-country validation or establish a common cross-country demand scale.

**Table S1. Achieved institutional and Impact Area participation.**

| **Completed questionnaires / respondent group** | **Nepal** | **Nigeria** |
| --- | --- | --- |
| All completed questionnaires | 27 | 28 |
| **Institutional affiliation (counts as reported)** | | |
| Public sector | 7 | 7 |
| Private sector | 7 | 1 |
| Academia/research | 2 | 8 |
| NGO/CSO | 3 | 2 |
| International development | 5 | — |
| International research | — | 9 |
| International aid/donor | — | 1 |
| Farmer organization | 1 | — |
| Other | 2 | — |
| **Impact Area entry (counts of routed respondents)** | | |
| Nutrition, Health and Food Security | 12 | 8 |
| Climate Adaptation and Mitigation | 7 | 10 |
| Poverty Reduction, Livelihoods and Jobs | 8 | 5 |
| Environmental Health and Biodiversity | 0 | 2 |
| Gender Equality, Youth and Social Inclusion | 0 | 3 |
| Notes: Institutional labels are retained as reported; — means not separately reported. Impact Area counts come from the selected route in Question 12 and sum to each country total. They are not denominators for every nested ranking. Small cells are descriptive; zero participation does not mean zero demand. Matched invitation counts, actor-family nonresponse and actor-by-module cross-tabulations are unavailable in these summaries, so response rates are not estimated. | | |

**Figure S1.** Nepal cross-cutting innovation priorities. Rank-weighted urgency of cross-cutting innovation and scaling domains (Nepal, country n = 27). Ranks 1–5 receive weights 5–1. Bar-end values are reported raw weighted totals, not percentages or normalized scores. This task is separate from Impact Area routing; Table S1 gives country and entry counts, not independently verified valid-case counts for this ranking task. The figure is reproduced from the country report [1] and is not used for numerical comparison between countries.

**Figure S2.** Nigeria cross-cutting innovation priorities. Rank-weighted urgency of cross-cutting innovation and scaling domains (Nigeria, country n = 28). Ranks 1–5 receive weights 5–1. Bar-end values are reported raw weighted totals, not percentages or normalized scores. This task is separate from Impact Area routing; Table S1 gives country and entry counts, not independently verified valid-case counts for this ranking task. The figure is reproduced from the country report [2] and is not used for numerical comparison between countries.

**Operational measures**

**Table S2. Reported operational measures for Nepal and Nigeria.**

| **Measure** | **Nepal** | **Nigeria** |
| --- | --- | --- |
| Submitted records | 27 | 28 |
| Reported routing inconsistencies | 0/27 (0%) | 0/28 (0%) |
| Submissions without informed consent | 0/27 (0%) | 0/28 (0%) |
| Invalid ranking options available in the tool | None | None |
| Reported missing or lost submission records | 0 | 0 |
| Reported device/power interruptions | 0/27 (0%) | 0/28 (0%) |
| Records retained for elapsed-time analysis | 27 | 28 |
| Median elapsed completion time, minutes | 77.5 | 45.7 |
| Interquartile range, minutes | 57.1–103.2 | 24.3–83.1 |
| Tool design and testing: estimated average effort per survey | 5 person-days; 40 hours | 5 person-days; 40 hours |
| Data curation and analysis: reported effort per country | 4 person-days; 32 hours | 4 person-days; 32 hours |
| Combined estimated effort for these activities | 9 person-days; 72 hours | 9 person-days; 72 hours |
| Notes: Values are reported operational summaries, not an independent audit of raw submissions or process logs. “None” for invalid options describes tool design, not an observed invalid-submission rate. Zero reported events do not rule out unrecorded pre-submission failures. Elapsed times may include pauses or save-and-return intervals. Effort uses an eight-hour person-day and covers only the listed activities. Eligible-item completion, invalid saved rankings, rejected attempts, cleaning exclusions and route-specific task burdens are not quantified in these summaries. Bangladesh participation and operational values are reported in main-text Tables 6 and 8. | | |

**Sources**

[1] Aravindakshan, S., Choudhary, D., Krupnik, T. J., Chakraborty, S., Kamal, M., Cofie, O., Marenya, P. P., & Martinez-Baron, D. (2026). Stakeholder informed innovation and scaling priorities in Nepal’s agrifood systems: A country profile report. CIMMYT. https://hdl.handle.net/10568/185460 (Sections 2.2 and 3.1–3.4; Figures 1, 2 and 4.)

[2] Aravindakshan, S., Krupnik, T. J., Chakraborty, S., Osei-Amponsah, C., Kamal, M., Choudhary, D., Cofie, O., Marenya, P. P., & Martinez-Baron, D. (2026). Stakeholder informed innovation and scaling priorities in Nigeria’s agrifood systems: A country profile report. CIMMYT. https://hdl.handle.net/10568/185461 (Sections 2.2 and 3.1–3.4; Figures 1, 2 and 4.)
